# Healthy and Osteoarthritis-Affected Joints Facing the Cellular Crosstalk

**DOI:** 10.3390/ijms24044120

**Published:** 2023-02-18

**Authors:** Sofija Semenistaja, Sandra Skuja, Anda Kadisa, Valerija Groma

**Affiliations:** 1 Department of Doctoral Studies, Rīga Stradiņš University, LV-1007 Riga, Latvia; 2 Joint Laboratory of Electron Microscopy, Institute of Anatomy and Anthropology, Rīga Stradiņš University, LV-1007 Riga, Latvia; 3 Department of Internal Diseases, Rīga Stradiņš University, LV-1007 Riga, Latvia

**Keywords:** osteoarthritis, synovial membrane, articular cartilage, subchondral bone, synoviocytes, chondrocytes, osteocytes, molecular signals

## Abstract

Osteoarthritis (OA) is a chronic, progressive, severely debilitating, and multifactorial joint disease that is recognized as the most common type of arthritis. During the last decade, it shows an incremental global rise in prevalence and incidence. The interaction between etiologic factors that mediate joint degradation has been explored in numerous studies. However, the underlying processes that induce OA remain obscure, largely due to the variety and complexity of these mechanisms. During synovial joint dysfunction, the osteochondral unit undergoes cellular phenotypic and functional alterations. At the cellular level, the synovial membrane is influenced by cartilage and subchondral bone cleavage fragments and extracellular matrix (ECM) degradation products from apoptotic and necrotic cells. These “foreign bodies” serve as danger-associated molecular patterns (DAMPs) that trigger innate immunity, eliciting and sustaining low-grade inflammation in the synovium. In this review, we explore the cellular and molecular communication networks established between the major joint compartments—the synovial membrane, cartilage, and subchondral bone of normal and OA-affected joints.

## 1. Introduction

Osteoarthritis (OA) is a chronic, progressive, highly debilitating, and multifactorial disease of a joint that is acknowledged as the most common form of arthritis, with an incremental rise in prevalence and incidence globally for the past decades [1]. According to the “Global Burden of Disease Study 2019” data, the burden of OA from 1990 to 2019 has increased by 113.5% and could be estimated at more than 500 million individuals [2]. About 10% of people worldwide suffer from OA-induced symptoms, such as functional disability, chronic pain, and mental health problems [3]. The prevalence of depressive symptoms among OA patients is estimated to be around 20% [3,4]. According to Shuang Zheng et al., depression is found in 25.4% of symptomatic knee OA patients with an annual incidence of 11.2% among them [5]. Han Lu et al. explored that there is a bidirectional association between depression and knee OA [6]. The progression of OA is deleterious to the quality of life, working capacity, health, and social welfare [7].

**Figure 1:**
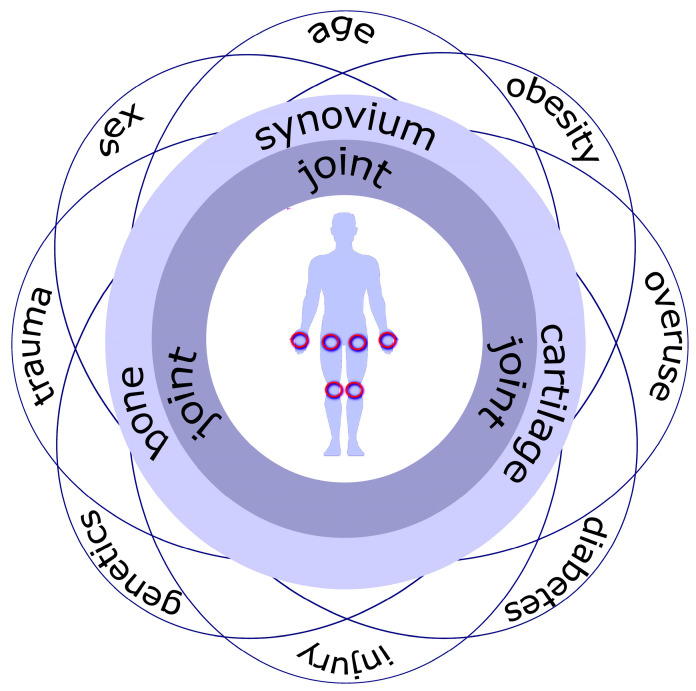
Common sites (red circles) and risk factors for OA: age, gender, genetics, metabolic state (obesity, type 2 diabetes), joint overuse, trauma, and injury that contribute to changes in the main joint compartments, namely, the synovial membrane, cartilage, fibrocartilage of meniscus, as well as subchondral bone.

Multiple factors are reported to contribute to the development of OA, such as increasing age, female sex, obesity and insulin resistance, joint mechanical overloading, experienced musculoskeletal trauma, and genetic predisposition [8] (Figure 1). 

Telomere length is a biomarker of aging that declines with advancing years, and its shortening is linked to the pathophysiology and development of OA [9]. In addition, senescent cells secrete factors that induce the aging of other cells in a paracrine manner [10]. Gao et al. found that senescence-associated beta-galactosidase expression by articular chondrocytes correlates with the severity of OA [11]. Evidence of gender differences in joint morphometry, kinematics, pain severity, use of healthcare resources, and functional recovery after arthroplasty is reviewed by Tschon et al. [12]. Obesity, defined as a body mass index greater than 30 kg/m^2^, more than triples the risk of knee OA [8]. According to Joseph et al., weight loss of more than 5% over four years is associated with less pain and slower knee OA radiographic progression on the Kellgren/Lawrence scale [13]. Joseph and colleagues explain these findings as a higher load transferred on the weight-bearing joint such as the knee or hip [13]. Furthermore, adipose tissue is a major source of adipocytokines, which modulate the immune response and inflammation both locally and systemically, thus contributing to joint degeneration [14]. Belluzzi et al. explored that changes in the infrapatellar fat pad (IFP) can predispose patients to OA progression by producing pro-inflammatory cytokines, growth factors, and profibrotic factors [15]. These changes in IFP facilitate prolonged pain in OA patients [16]. Wu and colleagues found that serum levels of adiponectin and leptin are negatively associated with bone mineral density in OA [17]. Type 2 diabetes and insulin resistance have been reported to associate with OA as a multifactorial pathology [18]. Mechanical load and post-traumatic injury both have been identified as undisputed risk factors in the development of OA; the latter is more common at a younger age and is associated with faster progression [19,20]. Recent research indicates that several OA genetic risk signals affect, or at least include, epigenetic regulators [21]. These risk factors create a beneficial background for a pathology. They are necessary, but not sufficient, to develop OA.

The primary attention in OA research thus far has been devoted to investigating joint cartilage [22]. The potential therapeutic approaches are oriented to stop or delay the progression of cartilage structural changes, thus reversing existing defects of the joint tissue [22]. At the same time, the latest available data about joint structural parts’ involvement in OA progression disclose the importance of research that highlights the interplay between components of a joint, respectively, subchondral bone, cartilage, and the synovial membrane. OA begins with joint dysfunction, primarily affecting the articular cartilage, synovium, and subchondral bone, all of which have close cellular and molecular interactions [23]. Moreover, in the case of a knee OA, the onset and progression of disease are strongly associated with meniscal lesions, both, posttraumatic and age-related degenerative [24,25].

Furthermore, even though, it is acknowledged that changes within joint tissue during OA are mainly degenerative, recent studies underline the importance of chronic inflammation [26,27,28,29,30,31,32,33]. The activation of the immune system, whether innate or adaptive, reflects local changes in the tissue. In the joint, it is strongly associated with low-grade systemic inflammation and the production of damage-associated molecular patterns (DAMPs) released during cartilage degradation [34,35,36,37,38,39,40]. Inflamed synovium acts as a trigger for OA progression, recruiting cells for intra-articular changes. To date, various cells have been recognized as potential contributors to synovial inflammation, mainly synoviocytes, macrophages, fibroblasts, and lymphocytes being the most prominent of them [41,42,43]. Activated cells trigger catabolic and pro-inflammatory cellular responses in the synovial membrane and fluid [44]. Finally, synoviocytes, being exposed to intracellular and extracellular DAMPs, activate chondrocytes and mediate the production of various matrix metalloproteinases (MMPs), chemokines, cytokines, and neuropeptides, thus establishing a vicious cycle between all joint tissue compartments [38].

In this review, we explore the cellular and molecular crosstalk established between the major joint compartments—the synovial membrane, cartilage, and subchondral bone of normal and OA-affected joints. 

## 2. Joint as an Organ in a Healthy State

**Figure 2:**
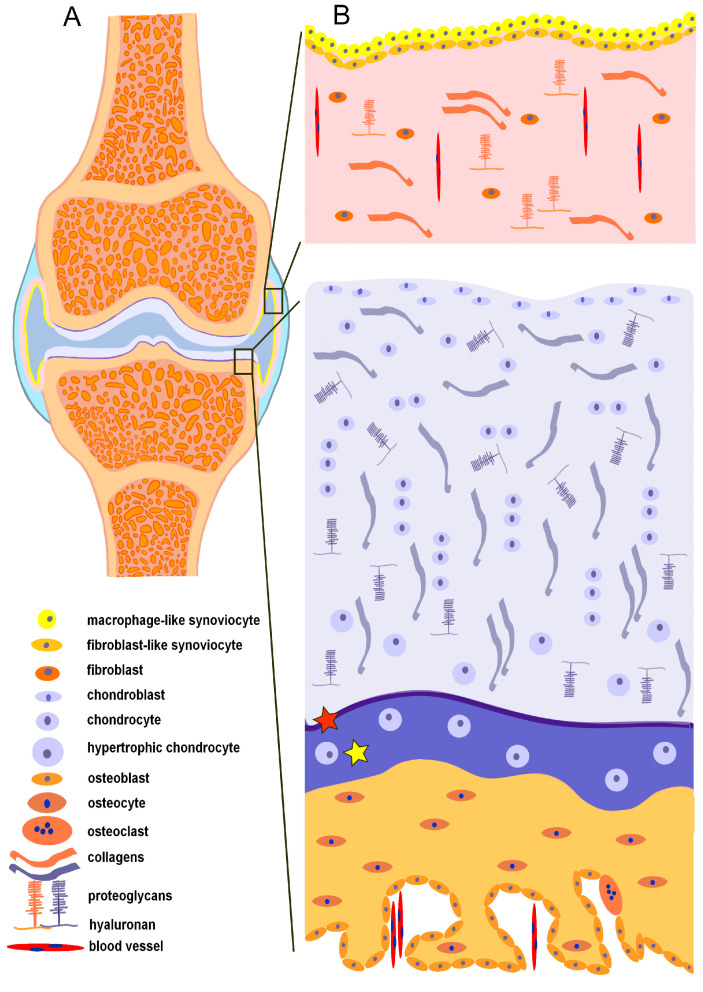
Schematic overview of a synovial joint structure. (**A**) Macroscopic view on the compartments of synovial joint. (**B**) The synovial membrane (upper part) in a healthy state. Composition of a healthy “osteochondral” unit (lower part), tidemark (red asterisk), and calcified cartilage (yellow asterisk).

The synovial joints represent the most common type of joint in the human body [45]. They are composed of subchondral bone, articular cartilage, and a two-layered synovial membrane, which surrounds all the mentioned elements, thus making the joint cavity filled with synovial fluid [45]. In physiological conditions, all joint-forming elements remain in balance or homeostasis [46] (Figure 2).

### 2.1. Synovial Membrane

The synovial membrane is the innermost portion of the joint capsule, which lines all non-articulating joint structures enclosing the cavity. It makes folded protrusions called villi in the joint cavity that increase the surface of the synovium, thus contributing to the joints’ adaptation during movements [47].

#### 2.1.1. Synovial Fluid

The synovial fluid is a viscous, transparent, and relatively hypocellular liquid. It is a blood plasma transudate that contains signaling factors (cytokines, enzymes, growth factors) and biomolecules in response to synovial fluid redistribution-induced sheer stress (hyaluronan, lubricin), which are synthesized by synovial membrane resident cells (mainly fibroblasts) [48,49]. The synovial fluid is the only source of nutrients for avascular, alymphatic, and aneural cartilage. Moreover, it facilitates lubricating properties, regulating biomechanical stress on the joint [49].

#### 2.1.2. Cellularity of the Synovial Membrane

The synovial membrane is composed of two layers: the lining (intima) layer and the sublining (subintima) layer. This delicate tissue represents a selectively porous barrier with intercellular junctions between the lining layer cells. It lacks the basement membrane that separates both layers [50]. On the one hand, it allows free passage for biomechanical, molecular, and cellular signals between synovial tissue and synovial fluid [51]. On the other hand, the synovial membrane has an immune regulatory function, providing homeostasis and integrity of the synovial tissue, adjacent cartilage, and subchondral bone [50,52,53]. The lining layer consists of two distinct cell types: type A and type B synoviocytes, also known as macrophage-like synoviocytes (MLSs) and fibroblast-like synoviocytes (FLSs). In healthy individuals, the synovial lining is two-layered. Close physical and chemical association and communication with synovial fluid are maintained by MLSs and FLSs [26]. The sublining layer is a loose connective tissue, which can be more adipose, areolar, or fibrous in type. This layer is supplied with blood vessels, lymphatic vessels, and nerves. The cellularity of the sublining layer is low. The major cell population is represented by resident cells such as fibroblasts and macrophages, and non-resident cells such as lymphocytes, and plasmocytes [48,54].

#### 2.1.3. Synovial Populations of Macrophages

The healthy synovial membrane niche contains several resident macrophage subsets based on their localization and surface markers [55]. In physiologic conditions, resident macrophages sense tissue damage, take part in the inflammatory response, and support tissue homeostasis. According to parabiotic mouse experiments and single-cell transcriptome sequencing, the healthy synovial membrane contains resident MLSs with Trem^2+^CX_3_CR1^+^ surface markers (phenotypic marker of efferocytosis MerTK^+^CD206^+^, subset Trem^2^, CX_3_CR1^+^ in humans) in the lining, accounting for 40% of the macrophage pool, resident interstitial MHCII^+^CSF1R^+^ macrophages (MerTK^+^Folr2^high^ID2^+^ in humans), and resident Lyve1^+^Relma^+^ perivascular macrophages (MerTK^+^Folr2^+^Lyve1^+^ in humans) in the sublining layer [55,56,57].

In the lining layer, MLSs are tissue-specific barrier-providing macrophages with “apical-basal” polarity, that form tight junctions (*zonula occludens*) and desmosomes, which thus determine the paracellular ion permeability. The main function of MLSs is to protect the adjacent sublining layer from biochemical stress that could be caused by cartilage degradation particles, cellular cleavage components, and pro-inflammatory cytokines, thus preventing spontaneous inflammation within joint tissue [50,52,58]. Unlike the lining layer, which has a single macrophage population (MLSs), the sublining layer is comprised of diverse resident macrophage populations. There are up to four distinct subsets of resident macrophages, classified according to the surface markers they express [50]. The origin of synovial resident macrophages remains obscure. They are blood monocyte-independent cells, and, theoretically, could appear prenatally [50,59]. Though, resident MLSs and perivascular macrophage pools can be restored from “precursor interstitial macrophages”: MHCII^+^CSF1R^+^ (MerTK^+^Folr2^+^ID2^+^ in humans) [59].

#### 2.1.4. Synovial Populations of Fibroblasts

Fibroblasts are mesenchymal-derived cells that can execute their lineage functions, such as accommodating the extracellular matrix (ECM) by synthesizing, assembling, and remodeling various types of collagen, proteoglycan, and fibronectin, as well as expressing MMPs and their inhibitors. Apart from coordinating remodeling processes, fibroblasts have resident tissue-specific functions [60]. FLSs are in charge of maintaining internal joint homeostasis. They do this by controlling the composition and turnover of synovial fluid, which lubricates and feeds cells in the deepest joint compartments. This function of FLSs is under the control of mechanical loading and inflammatory stressors [61]. An interaction of FLSs and MLSs supports the immunological barrier in a healthy synovial membrane [52]. Furthermore, all fibroblasts augment other cell functions in both healthy and affected tissue [58].

#### 2.1.5. Synovial Populations of Non-Resident Cells

Other cells that are found in the synovial membrane are vascular endotheliocytes, as well as immune cells such as lymphocytes, neutrophils, mast cells, plasmocytes, and dendritic cells. In healthy synovial membranes, most of them reside in small numbers in the perivascular compartment [62].

#### 2.1.6. Extracellular Matrix of the Synovial Lining

Resident fibroblasts are the main source of ECM. It is composed of fibrillar components such as collagens, fibronectins, elastin, and non-fibrillar components such as proteoglycans and hyaluronan. Cell-to-matrix interactions strictly coordinate the balance between the production, assembly, and degradation of ECM components [63].

### 2.2. Articular Cartilage

In synovial joints, articular cartilage is a highly specialized, load-bearing, friction-reducing connective tissue that overlays the ends of opposing articulating bone surfaces, providing smooth sliding for a joint during movements and executing properties of resiliency and deformability [46,57,64,65].

#### 2.2.1. The Composition of Articular Cartilage

In the recesses of a joint cavity, articular cartilage is attached to the synovial membrane that lines the joint. Cartilage is an aneural, alymphatic, and avascular tissue that receives nourishment from synovial fluid and the underlying bone [49]. Wang et al. explored that nutrition supplied by the synovial fluid is more significant for cartilage viability than that from subchondral bone [66]. Articular cartilage is made of chondrocytes that produce ECM, which is comprised of water (more than 70%), collagen, non-collagenous proteins, and proteoglycans [49,65,67]. The specific architecture of joint cartilage explains its limited ability to self-repair and its inherent limited healing potential. Unfortunately, both natural and pathological changes in articular cartilage may cause damage to the whole joint’s stability.

#### 2.2.2. Chondrocytes and Their Metabolic State

In mature cartilage, the arrangement of chondrocytes and ECM is more zonal [64]. The chondrocyte distribution, activity, and cellular shape, as well as the orientation and metabolism of the ECM structure within each zone, differ [64,68,69,70]. Histologically, there are four distinct layers in articular cartilage: the superficial, the middle, the deep, and the mineralized cartilage zones [69]. The latter zone is separated from non-mineralized cartilage by a tidemark [71]. In physiological conditions, the tidemark varies in number and thickness all along the cartilage. The tidemark plays a role in preventing the intrusion of mineralized cartilage into the non-calcified cartilage [72,73,74].

Up to 10% of cartilage tissue is occupied by chondrocytes, cells with phenotypic instability and poor regenerative capacity [64,67,68,75]. Chondrocytes are mechanosensitive cells whose cellular responses are influenced by their mechanical and chemical surroundings [61,76,77]. Normally, chondrocytes adapt their metabolic state to the environment by transducing received mechanical signals into chemical responses, which manifest as perturbations in gene expression responsible for remodeling processes, morphogenesis, the ECM, and matrix-degrading enzyme synthesis [78]. The synthetic ability of chondrocytes decreases with cartilage depth [79].

#### 2.2.3. Extracellular Matrix of Articular Cartilage

ECM is a complex network composed primarily of collagen type II (90% of all collagens), with minor pericellular collagens such as type IV, VI, and III, as well as fibril-associated collagens with interrupted triple helices (FACIT) collagens IX, XII, XIV, XVI, and XXI [67,80]. Collagen fibrils of articular cartilage ECM are arranged to resist the mechanical forces: collagen fibril orientation is parallel to the surface in the superficial zone, more randomly arranged in the middle zone, and vertically aligned in the deep zone [68,75]. That alignment is paralleled by chondrocytes’ arrangements and cellular activity profiles within each zone [81,82]. Thus, activated mature chondrocytes are mostly located in the superficial layer and partly in the middle layer; the cells synthesize collagen types II (COL2B), IX, and XI, whereas hypertrophic chondrocytes in the deep layer synthesize collagen type X [64,68,75]. Chondroprogenitor cells produce procollagen type IIA (COL2A) [82]. The pericellular matrix (PCM) buffers stress for chondrocytes and exhibits a different profile. It is mainly made of collagen types IV and VI, fibromodulin, and matrilin-3 [61,65,83]. Taken together, collagens provide articular cartilage with flexibility, compressive resistance, and tensile strength. Simultaneously, pericellular collagens anchor and maintain the integrity of chondrocytes, as well as mediate cell-to-matrix interactions [67]. The ECM collagen network is stabilized by proteoglycan aggregates, mainly aggrecan, with lesser amounts of decorin, biglycan, and fibromodulin, which make collagen type II inaccessible to proteinases and protect it from degradation, as well as non-collagenous proteins such as cartilage oligomeric matrix protein (COMP), link protein and many others [22,46,84].

### 2.3. Microenvironment in the Subchondral Bone

Together with articular cartilage, subchondral bone forms a morphofunctional unit called an “osteochondral unit” [85]. The osteochondral junction connects the calcified zone of the hyaline cartilage with the cortical plate of the subchondral bone and creates a mechanical and biochemical interplay between articular cartilage and subchondral bone [71,86]. The coupled mechanical interplay of both tissues increases the functionality of a joint, dissipating energy transfer [87].

#### 2.3.1. Architecture of the Subchondral Bone

Subchondral bone is separated from adjacent calcified cartilage by a cement line and is divided into two entities: subchondral cortical bone plate and subchondral trabecular bone [88]. The subchondral cortical bone plate contains channels with nerves and blood vessels that run from the subchondral trabecular bone. These blood vessels and nerves divide into smaller branches and reach into calcified cartilage. Subchondral trabecular bone is a sponge-like supportive structure that absorbs energy and supplies nutrients for remaining bone and cartilage [85].

Subchondral bone is a unique shock-absorbing tissue that dynamically adapts to the mechanical strain, decreasing the transmitted loading to the overlaying articular cartilage [88]. The process of adjusting bone structure to local strain is called “modeling”. The complex mechanism of bone resorption with subsequent formation is known as bone remodeling [89,90]. In physiological conditions, it is activated by biomechanical factors [91].

The microenvironment of subchondral bone depends on resident cells—osteoblasts, mature osteocytes, and osteoclasts—and their interaction with articular cartilage [71,85,92]. Lajeunesse and colleagues reported that prostaglandins, leukotrienes, and growth factors produced by subchondral osteoblasts could reach the calcified layer of articular cartilage [93]. Wu and his team found exosomes that osteoblasts release and thus facilitate intercellular communication between cartilage and bone [94].

#### 2.3.2. Osteoblast Lineage Cells

Osteocytes comprise 42 billion cells in the adult human skeleton with a cellular density of 19,000–28,500 cells/mm^3^ [95]. Their processes are embedded in the mineralized bone matrix and together with the lacunar–canalicular network, they make a connected system that links the vasculature with the mineralized surface, supplying the bone with nutrients [96]. Bone metabolism is mainly regulated through the wingless (Wnt) signaling pathway and the receptor activator of nuclear factor kappa B (NF-κB)/receptor activator of NF-κB ligand-osteoprotegerin (RANK/RANKL-OPG) pathway [97].

Osteoblast lineage cells release bone metabolic activity regulator proteins such as sclerostin, RANKL, and OPG [98]. RANKL and OPG are mainly expressed by osteoblasts. Osteocytes are the major source of skeletal sclerostin, an inhibitor of the wingless-related MMTV integration site’s (Wnt) canonical signaling pathway. The Wnt signaling pathway induces mesenchymal stem cell differentiation into osteoblasts [99]. Song et al. explored that forced Wnt7b expression in mice results in high bone formation [100]. Sclerostin synthesis is mainly amplified by immobilization [101,102]. Skeletal sclerostin gene knockout mice demonstrate high bone mass [103]. The induction of sclerostin synthesis is a major factor in stopping osteoblast-associated bone remodeling [104].

Osteoblasts are responsible for extracellular bone matrix synthesis, as well as for osteoclastogenesis by RANKL and OPG expression as well as the canonical Wnt signaling pathway [105].

#### 2.3.3. Macrophage Lineage Cells

Osteoclasts are macrophage lineage cells that are responsible for bone resorption. RANKL is a positive regulator of osteoclastogenesis [106]. OPG is a soluble decoy receptor that possesses an antagonizing effect, neutralizing RANKL and maintaining the bone [107].

### 2.4. Cellular and Molecular Interactions in the Healthy Joints

The cross-tissue interaction in subchondral bone and cartilage and a network-based molecular regulation by the synovium are responsible for healthy environments in joints [24,108]. In Table 1, we collected and represented data focusing on a given healthy condition—molecular activity in each resident or non-resident cell type.

## 3. Pathological Changes in OA-Affected Joint Tissues

**Figure 3:**
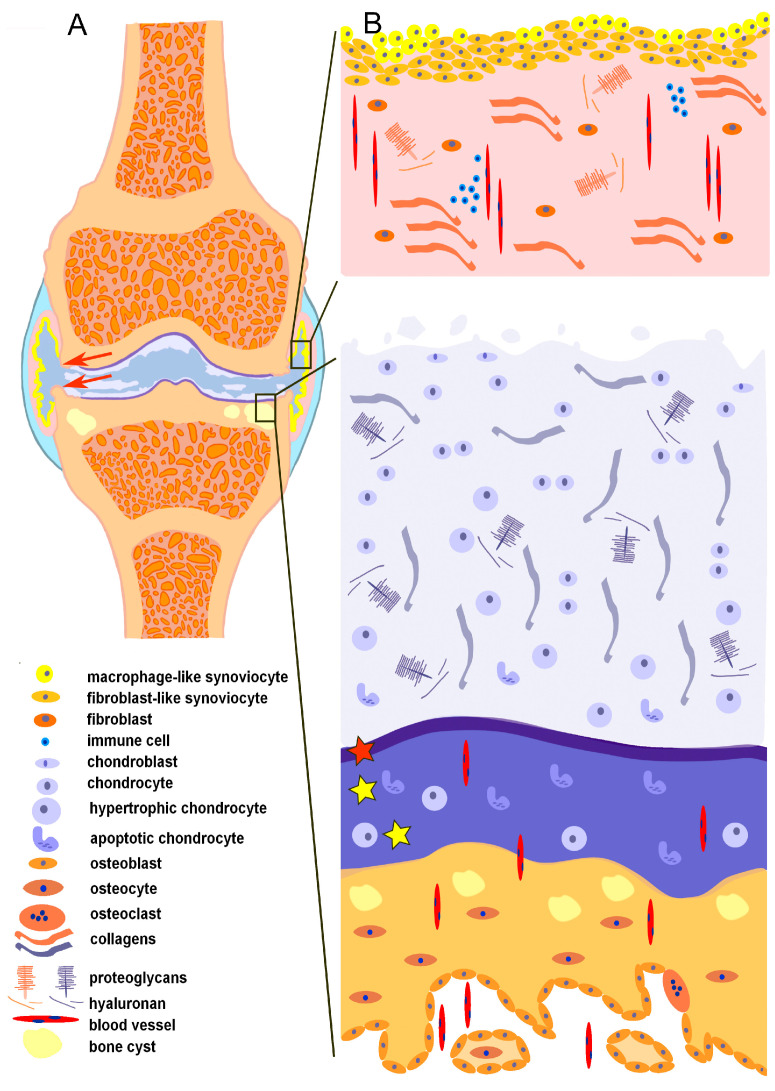
Schematic overview of the OA-affected synovial joint with involvement of both “chondrosynovial” and “osteochondral units”. (**A**) Synovial joint depicting common changes, such as degradation of the articular cartilage, formation of subchondral bone cysts, and osteophytes (red arrows). (**B**) The synovial membrane (upper part) reveals hyperplasia of the lining layer, immune cell infiltration, angiogenesis, and fibrosis in the sublining layer. Alterations of the articular cartilage (lower part) include degradation of the non-calcified zone and thickening of the calcified zone (yellow asterisks), formation of free cartilage fragments, duplication of tidemark (red asterisk), alterations in the composition of cellular and extracellular matrix. Remodeling of the subchondral bone (lower part) reveals the thickening of the cortical bone plate, formation of cysts, and angiogenesis.

OA has been described for a long time as a mechanically induced degenerative process of the joint [78,114]. OA starts gradually when the disturbance in the joint microenvironment appears with an imbalance between synthesis and destruction processes, pro-inflammatory and anti-inflammatory effects [115]. The pathological processes result in cartilage loss, subchondral bone remodeling, and synovial inflammation (Figure 3). The main joint components—synovial membrane, articular cartilage, and subchondral bone—contribute to the development of OA to varying degrees, thus reflecting various OA subgroups and phenotypes in patients [116].

### 3.1. OA-Affected Synovial Membrane Triggers and Perpetuates the Pathological Process

There is increasing evidence that the synovial membrane plays the role of “a cornerstone” in the progression of OA. OA presents with pain, joint swelling, and morning stiffness, which are mostly linked to synovial inflammation (synovitis) [40,117]. In combination with mechanical factors, synovitis could be the first trigger for the activation of the immune system and the further perpetrator of the pathologic cycle in OA pathogenesis [118]. The importance of synovitis in OA progression, joint destruction, and associated manifestations has been highlighted over the last decades [119,120].

#### 3.1.1. Changes in the Biochemical Profile of Synovial Fluid in OA

Despite the low number of immune cells found in joint tissues, serum, and synovial fluid in OA patients, the alterations in synovial fluid’s biochemical profile reflect changes in cell metabolism in the joint. Under the influence of joint disease, many cytokines and collagen turnover biomarkers accumulate in synovial fluid, including COMP, CC-chemokine ligand 5 (CCL5), matrix metalloproteinase-1 (MMP-1), MMP-3 and MMP-18, as well as tumor necrosis factor-alpha (TNF-α), interleukin-6 (IL-6) and IL-8 [121]. Elevated IL-6 level in synovial fluid may be a predictor of severe OA, and its level correlates with severe pain and is associated with chronic inflammation, while the concentration of COMP found in the serum correlates with the number of OA-affected joints [7].

#### 3.1.2. Alterations in of the Synovial Membrane Reflecting Inflammatory and Fibrotic Changes

The microenvironment of the synovial membrane undergoes substantial changes in the case of joint inflammation. Hyperplasia of the lining layer and increased cellular density of the sublining layer, inflammatory cell infiltration (macrophages, T and B cells), and angiogenesis are all common features of OA-related synovitis [122]. Krenn et al. proposed a histopathological grading system to evaluate the damage to the synovial membrane and define the synovitis grade in inflammatory and non-inflammatory arthritides by using light microscopy [54]. Patients with OA-related synovitis may have different levels of inflammation, but in most cases, it is a low-grade [26,27].

Synovitis occurs in more than 50% of early OA patients [33,48]. It is linked to a higher risk of cartilage damage, thus worsening the disease, and correlates with an increase in pain sensation [120,123,124,125,126,127]. According to arthroscopic and soft tissue ultrasound (US) examination data, synovitis, confirmed by these imaging methods, is seen before OA is revealed in conventional radiographs and cartilage damage is present in magnetic resonance imaging (MRI) [120,128]. Thus, it could be concluded that synovitis could precede cartilage damage or the development of radiographic OA. Riis et al. found a positive correlation between MRI data, cartilage histopathological data, and the severity of synovitis, whereas Abbasi and colleagues revealed a correlation between the severity of ultrasonographic synovitis and pain [129,130]. Synovitis increases the risk of presenting with painful knee OA ninefold [124]. Zhang and colleagues explored that pain in OA patients is positively associated with synovitis and bone marrow lesions (BMLs). The diminution of BMLs and synovitis frequently results in pain resolution [131]. However, Bacon et al. found that a 0.1 mm decrease in cartilage thickness over 2 years increases the Western Ontario and McMaster Universities osteoarthritis index (WOMAC) pain score by only 0.32 points, implying that cartilage loss does not significantly worsen the pain and that the cartilage loss associated with pain is mainly mediated by synovitis [132].

Synovial membrane cells produce mediators with proteolytic and pro-inflammatory activity [33,65]. Approximately 55% of OA-related cytokines are produced by synoviocytes; 38% of them are produced exclusively by synoviocytes [33]. Elevated levels of pro-inflammatory mediators are found in the synovial fluid, synovial tissue, and serum of OA patients [133,134,135]. Nonetheless, all findings from controlled randomized trials on the potential use of biological agents in OA treatment remain controversial, with no significant differences in OA alleviation compared to a placebo [136]. Unfortunately, as the grade of synovitis differs between OA subjects, the synovial membrane’s cellular compartment is highly heterogeneous between patients, thus the dominant effector cell type in OA pathogenesis remains unknown [33,57]. Chou and colleagues performed single-cell RNA sequencing, revealing 12 distinct cell subpopulations in the OA synovium, more than 77% of them being FLSs, and sublining fibroblasts and about 12% being HLA-DRA^+^ cells, including macrophages, dendritic cells, proinflammatory fibroblasts, and B cells [33]. Macrophages, fibroblasts, and other cellular players can impact each other’s transcriptomes, propagating synovial inflammation, cartilage breakdown, and subchondral bone sclerosis [33].

Synovial fibrosis is another OA manifestation related to joint pain and stiffness [137]. It is a consequence of chronic inflammation and angiogenesis. Synovial fibrosis is caused by an imbalance between collagen synthetic and proteolytic processes as a response to pro-inflammatory factors such as IL-6, IL-1β and pro-fibrotic factors such as transforming growth factor-beta (TGF-β) and connective tissue growth factor (CTGF). MLSs and FLSs are both implicated in synovial fibrosis [138,139]. Interestingly, IFP has been acknowledged as a source of pro-inflammatory factors and adipokines, thus potentially contributing to synovial inflammation and fibrosis [140]. Furthermore, the IFP function is thought to be linked to the synovial membrane [141]. Both can contribute to fibrosis, hypervascularity, and vascular remodeling, resulting in a pro-inflammatory microenvironment, as well as pain sensitization [141,142]. Moreover, Belluzzi et al. highlighted an elevated expression of Piezo1/2 mechanosensitive ion channels in IFP and synovial membrane vasculature that suggests the aforementioned contribution of this anatomo-functional unit to pain in OA [143].

#### 3.1.3. Insights into Synovial Macrophage Diversity Confirmed in OA Patients

Resident and non-resident synovial lining and sublining macrophages are the most prominent cellular component of innate immunity in OA [42]. One of the classical ways to distinguish macrophages is by their state of polarization. The cells are classified as M1 (pro-inflammatory) or M2 (anti-inflammatory) depending on the surface markers. This concept of M1 and M2 macrophage polarization exists since Mills et al. defined two activation poles as M1 and M2 in 2000 [144]. However, a recent study suggests that there are at least three subsets of M2 macrophages: M2a, M2b, and M2c [59]. M2 macrophages contribute to tissue repair and remodeling by releasing IL-10, TGF-β, IL-1 receptor antagonists, and a variety of other mediators [49,57,115]. The transition from M2 to the M1 state accelerates joint destruction via TNF-α, IL-1β, IL-6, and IL-17 [57]. As macrophages have even greater phenotypic heterogeneity and plasticity, characterized by the presence of immunoregulatory and cartilage remodeling proliferative functions, this conception is now considered extreme and imprecise [33].

Upon the onset of synovitis, blood-derived macrophages (MerTK^−^) infiltrate the sublining layer, leading to the formation of a variety of pro-inflammatory non-resident macrophage subtypes [58]. Sublining inflammatory macrophages produce more pro-inflammatory mediators than lining MLSs [33]. Activated macrophages are associated with cartilage breakdown directly and indirectly via the secretion of cytokines and the dynamic interplay between fibroblasts and chondrocytes. They produce MMP-1, MMP-3, MMP-9, MMP-13, and aggrecanase and downregulate anabolic processes within cartilage [57]. Blom et al. identified that macrophage depletion from the synovial membrane causes a significant decrease in MMP-3 and MMP-9 [145]. The abundance of activated macrophages in the sublining layer correlates with knee OA radiographic severity and symptoms [146]. Huo et al. found elevated levels of the macrophage chemoattractant fractalkine (CX_3_CL_1_) and CCL2 in OA patients’ synovial fluid and serum as well as their positive association with pain and poor clinical outcome [147]. Interaction with CX_3_CL_1_ induces pro-inflammatory and degrading enzyme production by macrophages [148]. In animal models, CCL2/CCR2 blockade results in macrophage number reduction, diminished synovitis, and cartilage damage [149]. According to Daghestani et al., soluble macrophage-associated molecules sCD163 and sCD14 shed from activated macrophages, are found in OA synovial fluid and serum, and reflect OA symptomatic severity, osteophyte formation, and joint space narrowing [150]. In another study, macrophage depletion from the synovial membrane was associated with a reduction in osteophyte formation during experimental OA. Downregulation of both TGF-β and bone morphogenetic protein-2 (BMP-2) plays a role in osteophyte formation in this case [151].

Over time, mechanical and biochemical factors reduce the abundance of MLSs (MerTK^+^CX_3_CR1^+^Trem2^+^ lining macrophages) from 40% to 10% of all synovial macrophage pools. This results in tight junction disruption between MLSs and the breakdown of the lining layer’s barrier-like structure. According to MRI data, the breakdown of the MLS barrier-like structure is associated with increased intra-articular accumulation of the infused contrast agent [50]. In the arthritic mouse models, the depletion of these cells leads to increased inflammatory cell infiltration into the synovial membrane and a pro-inflammatory microenvironment [50,52,57]. Of note, in inflammatory arthritis, MerTK^+^ macrophage number inversely correlates with disease activity, angiogenesis, and hyperplasia of the synovial membrane [55].

#### 3.1.4. Distinct Subsets of Synovial Fibroblasts Confirmed in OA Patients

Inflammatory and mechanical factors influence the phenotype and function of FLSs and sublining fibroblasts [152]. The amount of FLS-synthesized lubricin and hyaluronan is decreased in the OA joint under the influence of excessive stress and pro-inflammatory cytokines [152]. This accounts for the diminished volume and viscosity of synovial fluid that is associated with OA-related pain. Interestingly, intra-articular lubricin injections into OA mouse models downregulated the inflammatory environment and alleviated pain [153]. Intra-articular hyaluronan injections may also provide pain relief and improve joint function [154]. Noteworthily, the diminished hyaluronan synthesis by FLSs is attributable to the pro-inflammatory microenvironment in OA, which induces the depolymerization of high molecular weight hyaluronan into small oligosaccharides [155]. FLSs and sublining fibroblasts’ phenotypes switch to destructive and pro-inflammatory, respectively. However, there is no clear evidence for a phenotype switch in OA when compared to typical inflammatory arthritis [156,157]. Nevertheless, some studies describe the activation of synovial fibroblasts with upregulation of the NF-κB pathway under the influence of pro-inflammatory factors [158]. A switch from FLSs to myofibroblast-like cells is associated with synovial fibrosis [159]. Moreover, elevated levels of fractalkine CX_3_CL_1_ in the synovium induce FLSs and sublining fibroblasts to synthesize MMP through the NF-κB pathway, particularly MMP-3, MMP-9, MMP-2, which degrade aggrecan and collagen [148,160,161]. Studies on transcriptomics reveal that all synovial fibroblasts demonstrate positional diversity, which makes them respond differently to the same pro-inflammatory signals. As a result, the TNF-α response may result in matrix degradation or an increase in pro-inflammatory responses [162].

#### 3.1.5. Non-Resident Cell Role in the Progression of OA

Trajerova and colleagues identified several immune cell phenotypes of knee OA based on synovial fluid-derived immune cell composition. They found increased infiltration of T cells, monocyte–macrophage lineage cells, NK cells, and neutrophils [163]. A flow cytometry analysis showed the higher infiltration of synovial fluid and synovial tissue by CD4^+^ T cells with an increased CD4^+^ to CD8^+^ ratio, particularly Th1-polarized cells, in early stage and end-stage OA [43,164]. In addition, the abundance of Treg cells is usually reduced [118].

#### 3.1.6. Danger-Associated Molecular Associated Patterns Characteristic of OA-Caused Damage

The immune system is an active player during synovitis. Immune cells react to mechanical and biochemical stimuli that accumulate within joints over time [165]. Schedel and colleagues found that cartilage and subchondral bone cleavage fragments, and ECM degradation products stimulate the synovial membrane. This stimulation results in FLSs and MLSs binding to debris with further absorption of them, producing detritus synovitis. These changes do not lead to an invasion, as it is in RA [166]. The formation of detritus synovitis may have a further aggravating effect on the inflammation process as well as on cartilage loss [166]. Thus, it can be speculated that cartilage and subchondral bone ECM components could stimulate the innate immune system [7]. In that case, ECM components and intracellular proteins from apoptotic or necrotic cells, which occur after prolonged mechanical overuse or during the physiological aging process, are considered foreign bodies. As foreign bodies, they serve as DAMPs that trigger innate immunity, eliciting and sustaining sterile low-grade inflammation [57,117,167,168].

DAMPs or alarmins are endogenous molecules produced during tissue remodeling that link inflammation with immune responses in OA [169]. Physiologically, they induce immune responses to trigger the repair of damaged tissue and the clearance of debris [117]. In OA, this response is altered [117]. ECM cleavage remnants from proteoglycans, fibronectin, low-molecular-weight hyaluronan, tenascin C, intracellular cleavage products from apoptotic or necrotic cells, uric acid, and other crystals, as well as plasma proteins, serve as DAMPs [170,171]. DAMPs, S100A8 and S100A9 proteins, and uric acid are closely related to the age-related OA phenotype [171]. Depolymerized hyaluronan fragments accumulate within the synovial membrane and upregulate membrane-associated Toll-like receptor 4 (TLR4) and CD44 receptors’ expression [172]. Interestingly, cell migration-inducing hyaluronidase-1 (CEMIP), which plays a role in hyaluronan catabolism, is increased in inflamed synovial membranes and overexpressed by FLS [173]. Moreover, the DAMP-like action of hyaluronan is molecular weight-dependent [172]. Low-molecular-weight hyaluronan and small oligosaccharides propagate further degradation of the ECM and amplify the inflammation responses through the NF-kB pathway, thus stimulating the release of pro-inflammatory mediators and destructive enzymes [172].

Calcium-containing crystals, both basic calcium phosphate (BCP) and calcium pyrophosphate dihydrate (CPP), are potential DAMPs that are found in the synovial fluid of OA patients, induce pro-inflammatory responses in synovial tissue, and exacerbate articular cartilage calcification [174,175].

DAMPs are recognized by pattern recognition receptors (PRR), which include TLRs on the surface of immune cells, cytoplasmic PRRs such as nod-like receptors (NLRs), or secreted receptors such as complement receptors [49,176]. TLRs are found on the cell surface (TLR-1, TLR-2, TLR-4, TLR-5, TLR-6) and on the surface of endosomes (TLR-3, TLR-4, TLR-7, TLR-8, TLR-9). Although in smaller numbers than in RA, TLR-2 and TLR-4 are found to be overexpressed in the OA synovial membrane [165]. Stolberg-Stolberg and colleagues studied the contribution of TLR-3 activation to cartilage degeneration. In vitro, it was revealed that TLR-3 is upregulated in chondrocytes of OA cartilage and reacts to apoptotic chondrocytes. The TLR-3 knockout mice were protected from OA-like cartilage breakdown [177]. It is also worth mentioning, that satellite glial cells of the dorsal root ganglia (DRG) express many TLRs. Miller et al. revealed that TLR-2 in the DRG is activated by aggrecan cleavage fragments, promoting OA-associated pain [178]. TLRs activation triggers the production of pro-inflammatory cytokines, chemokines, proteolytic enzymes, and growth factors such as TNF-α, IL-1β, IL-6, IL-8, IL-15, IL-17, IL-18, IL-21, CCL-5, MMP-1, MMP-3, MMP-9, MMP-13, TGF-β, fibroblast growth factors (FGF), vascular endothelial growth factor (VEGF), nerve growth factor (NGF), and many others by activation of NF-κB, activator protein-1 (AP-1), and mitogen-activated protein kinase (MAPK) [117,165,170]. In addition, the complement system may be implicated in endogenous molecules that induce chronic inflammation in OA [117].

### 3.2. Degradation of the Articular Cartilage in OA

The changes to articular cartilage include alterations to the cartilage matrix and cellular components. Disturbed matrix cellular organization leads to tissue fibrillation, vertical fissures, degradation of the cartilage, and endochondral ossification. On a molecular level, the loss of aggrecan, decreased quantity and quality of collagen type II, collagen type I accumulation, and perturbations of chondrocytes are observed [114]. Overall, it results in decreased tensile strength and energy-storing capacity, limiting joints’ functional abilities.

#### 3.2.1. Chondrocyte Phenotypes in Cartilage Affected by OA

Compositional and organizational changes in ECM and pericellular matrix (PCM) affect chondrocytes’ behavior [67,179,180]. Normally quiescent chondrocytes start clustering to form multicellular chondrons to satisfy anabolic demands [167]. Unfortunately, the composition of newly produced collagen is switched from type II to type I, which weakens cartilage durability and turns hyaline cartilage into a tensile-incompetent fibrotic cartilage-like structure [75,181]. Even though with time, actively proliferating, swollen chondrocytes become more susceptible to hypertrophy and dedifferentiation, it inevitably results in cellular damage and death [182]. In the late stages of OA, chondrocyte anabolic activity cannot compensate for catabolic changes within cartilage, leading to a thin, calcified, and functionally incompetent structure that propagates into the non-mineralized cartilage, reducing its thickness [45,82].

Chondrocytes undergo phenotypic changes in response to mechanical stress, various self-produced cytokines, and growth factors, accompanied by the synovial membrane and subchondral bone cell-produced pro-inflammatory factors. Chou and colleagues explored the phenotypic and functional heterogeneity of articular chondrocytes and their potential upstream regulators during OA by genotyping [33]. They identified seven distinct cell phenotypes such as homeostatic chondrocytes (HomC), prehypertrophic chondrocytes (preHTC), hypertrophic chondrocytes (HTC), regulatory chondrocytes (RegC), prefibrochondrocytes (preFC), fibrochondrocytes (FC) and reparative chondrocytes (RepC). PreFC, FC, preHTC, RepC, and RegC are predominantly seen in the arthritic part of the cartilage. HomC regulates cell metabolism, development, and homeostasis [33,183]. PreHTC expresses genes to control hypertrophic differentiation [183]. HTC expresses genes to control ECM mineralization but also induces angiogenesis and osteoblast invasion into cartilage tissue [183]. RegC expresses genes for antigen-presenting function, as well as modulates different signaling pathways [183]. RepC expresses genes for ECM remodeling, while PreFC and FC express genes for fibroblast-like activity [33].

However, there are studies suggesting the presence of more chondrocyte phenotypes [68,184]. A dedifferentiated chondrocyte is another phenotype of a cartilage cell. In physiological conditions, the microenvironment and other factors such as the master regulator SRY-box transcription factor 9 (SOX-9) restrain chondrocytes from losing their physiological chondrogenic phenotype or dedifferentiation [185]. Morphologically and functionally, dedifferentiated chondrocytes become fibroblast-like cells, which despite their high synthetic ability, produce collagen with reduced mechanical properties (collagen type I, type III, type V), thus contributing to articular cartilage disturbance, remodeling, and fibrosis [64,82]. Synoviocytes also release pro-inflammatory cytokines, such as IL-1α, IL-1β, and TNF-α, which aid in dedifferentiation. Moreover, dedifferentiation also leads to a hypertrophic osteoblast-like phenotype with increased MMP-13 expression in chondrocytes and collagen type X secretion. Nidogen-2 is one of the chondrocyte pericellular matrix components, that regulates the antagonizing action of RUNX2 and SOX-9. Nidogen-2 depletion also contributes to the hypertrophy of chondrocytes and cartilage calcification [186]. As mentioned above, IFP is a major intra-articular source of pro-inflammatory cytokines or adipokines. Gomez and the team reported that adiponectin induces the expression of IL-8 in chondrocytes more potently than IL-1β, thus contributing to the degradation of articular cartilage [187].

Furthermore, available data suggest that chondrocytes express a resistant-to-apoptosis senescent cell phenotype with a proinflammatory secretome in damaged cartilage [188,189,190]. Moreover, senescent chondrocytes are present in osteoarthritic lesions with an increased tendency to accumulate with age but are absent in intact cartilage [189,191]. Jacob and colleagues explored that chondroprogenitor cells obtained from OA cartilage display morphological features of senescent cells, expressing a senescence-associated secretory phenotype with increased levels of reactive oxygen species (ROS), IL-6, and IL-8 [192]. In addition to that, the authors report the main stress-inducers, metabolic changes with impaired mitochondrial function, oxidative stress, and genomic damage with upregulated p16^INK4a^ expression [167,192,193].

#### 3.2.2. Remodeling of Extracellular Matrix during the OA

Cartilage swelling is the first sign of the ECM degradation process, which appears when the osmotic properties of cartilage are changed [194]. It is detected by MRI as cartilage “thickening” and positively correlates with proteoglycan loss in early experimental arthritis [195]. Bank et al. first explored that swelling of the cartilage appears due to the loss of the collagen network [196]. Other studies have highlighted that increased catabolism of proteoglycans, particularly aggrecan, is the main contributor to the increase in water content in cartilage tissue with no significant differences in collagen composition in early stage OA [197,198]. On the molecular level, cartilage hyperhydration is driven by the loss of aggrecan’s G1 domain, which normally binds this proteoglycan to the link protein, hyaluronan, and matrix itself [49,199]. Interestingly, the loss of hyaluronan from cartilage and its depolymerization is also associated with pro-inflammatory conditions and enhanced production of IL-1β and TNF-α that lead to CEMIP overexpression by chondrocytes correlated with OA activity [173]. The perturbations in the composition of the proteoglycan network typically begin in the superficial zone of the cartilage extending to the deep zone as the OA evolves [200]. It is worth mentioning that aggrecan degradation by itself does not lead to OA progression. Major proteoglycan degradation proceeds with the loss of minor ECM components, an irreversible breakdown of microfibrillar collagen, and the depletion of collagen type II, resulting in disruption of the collagen network, known as fibrillations and fissures [46,199].

The changes in ECM influence the composition of PCM [61]. PCM is a part of ECM, which surrounds the groups of chondrocytes, making a common entity called the chondron. Disturbed integrity of PCM is associated with the loss of microfibrillar collagen type VI, the main component of the PCM, as well as collagen type IV, percelan, fibronectin 1, nidogens, and laminins [201]. Schminke et al. found that chondroprogenitor cell stimulation by nidogen-2 stimulation decreases Runt-related transcription factor 2 (RUNX2) and increases both SOX-9 mRNA and aggrecan, whereas stimulation by laminin upregulates type II collagen synthesis and downregulates type I collagen synthesis, thereby supporting chondrogenesis. Thus, the authors conclude that the decrease in PCM components, as it is observed in OA, contributes to the increase in collagen type I and cartilage endochondral ossification [186].

It is reported that the loss of microfibrillar collagen type VI alters the mechanotransduction through the chondrocytes’ primary cilium [202]. Furthermore, PCM collagen type IV depletion aids to change the viability and phenotype of chondrocytes [67,78,203].

Overall, PCM alterations decrease chondrocyte abilities to sense mechanical signals, exposing the cell to swelling, and making them susceptible to phenotypical perturbations.

#### 3.2.3. Cartilage Destruction-Associated Proteases Activated in OA

All catabolic changes within articular cartilage are managed by excessive production of matrix-degrading enzymes, such as matrixins or zinc-dependent endopeptidases, MMPs, and a disintegrin and MMP with thrombospondin motifs (ADAMTS), as well as a diminished synthesis of their inhibitors [204,205]. The key aggrecan-degrading enzymes are ADAMTS-4, ADAMTS-5 and ADAMTS-9, to a lesser extent MMP-1, MMP-3, MMP-9, MMP-13 [206]. The major structural macromolecule in the ECM, collagen type II, is cleaved mainly by MMP-13, MMP-1, and MMP-3. These matrix-degrading enzymes, which act in an autocrine and paracrine fashion, are synthesized by both chondrocytes and synovial FLS [31,45]. MMP hyperproduction and hyperactivity, particularly ADAMTS, are caused by altered post-translational endocytosis of these enzymes [207,208]. Genetic polymorphism in the enzyme that regulates ADAMTS activity, as well as in the enzyme structure, is positively associated with the occurrence of OA [208,209]. OA-related proinflammatory cytokines, which are derived from synovial membrane cells, also contribute to the hyperproduction of matrix enzymes [207].

#### 3.2.4. Cartilage Destruction-Associated Cytokines Contributing to OA Development

A major part of the OA key cytokines that regulate chondrocyte transcription and function originate from synovial membrane resident cells; 38% of OA-related key cytokines are exclusively produced by synoviocytes and none of the cytokines are exclusively produced by chondrocytes [33]. According to recent studies, resident macrophages and non-resident dendritic cells, but not chondrocytes, express interleukin-1 beta (IL-1β), interleukin-6 (IL-6), and TNF-α, all of which are mostly expressed in synovial membranes [33]. ADAMTS-5 expression is mainly activated by IL-1β, TNF-α, and TGF-β [210]. Notably, MMP-13, the main collagen type II cleaving enzyme, is expressed not only by chondrocytes but also by FLSs and osteocytes, thus pointing to a relationship between joint cells and their increased impact on cartilage [7]. Anabolic changes are stimulated by TGF-β and insulin-like growth factor-1 (IGF-1). Catabolic changes are mostly controlled by IL-1α and IL-1β [33,211]. Cai and the team have studied that chondrocyte stimulation with IL-1β elicits an immune responsive gene 1 (IRG1) associated pro-inflammatory response [212].

### 3.3. Alterations in the Subchondral Bone Affected by OA

The microstructure of the subchondral bone changes during the OA progression. Changes in cortical bone plates: volume, porosity, composition, and mineralization degree; transformation of the trabecular bone compartment; the appearance of bone marrow lesions (BMLs); neovascularization; cyst and osteophyte formation; and sclerosis are examples of typical alterations [45,213]. When the bone’s adaptive capability to mechanical and biological signals is exceeded, bone remodeling decouples [48,214].

#### 3.3.1. Microstructure of Subchondral Bone during the Early Stage OA

In the early stages of OA, a high bone turnover with resorption dominates, resulting in cortical bone thinning, increased porosity, trabecular compartment widening, and separation of trabeculae [22,88]. Findings of high bone turnover are supported by the hyperexpression of bone resorption markers [46,91]. For example, Huebner et al. found that the urinary alpha C-telopeptide of type I collagen (α-CTX) marker, which is a metabolite of type I collagen, was elevated in regions with newly formed bone as confirmed by scintigraphy, corresponding to the sites of high bone turnover in knee OA [215]. According to Zhao et al., high bone turnover is reflected by increased rates of leukemia inhibitory factor (LIF), a cytokine secreted by osteoclasts that upregulate the Wnt signaling pathway by lowering sclerostin in osteocytes [91]. The intensity of LIF expression correlates with the stage of OA evolution [216].

#### 3.3.2. Microstructure of Subchondral Bone during the Late-Stage OA

In the late stages of OA, increased bone synthesis and hypomineralized bone volume are more evident [85]. The relative hypomineralization of bones is explained by the discordance of still high bone turnover and mineralization processes [46]. The cortical bone plate becomes thicker and the rod-to-plate ratio in trabecular bone decreases [88,89,213]. The hallmarks of late OA are osteoid islets and sclerosis [85]. BMLs are one of the results of abnormal reciprocity between cartilage and subchondral bone and are associated with pain and disease progression [217,218]. BMLs occur before the development of radiographic OA and are observed by MRI [218]. BMLs, as well as subchondral bone cysts, express a positive correlation with synovitis severity. BMLs, once present, tend to increase [45,48].

#### 3.3.3. Changes in the Cellular Composition of the Subchondral Bone

On the cellular level, the changes observed are orchestrated by the subchondral bone resident cells, their interaction with chondrocytes and synoviocytes, and the supervision of mechanical and biological signals [214].

In the early stages of OA, osteocytes increase the expression of RANKL and sclerostin and decrease the expression of osteoprotegerin (OPG), thus increasing bone resorption [214,219]. Over the course of the disease, osteocytes shift their phenotype to OA-associated, meaning round-like shape and disarrangement, which interferes with their ability to sense and transduce mechanical stimuli [220].

Impaired osteocyte perilacunar remodeling (PLR) is both the initiator and consequence of OA degenerative processes within the subchondral bone and articular cartilage [221]. Mazur and colleagues found that a deficiency of PLR in OA, particularly MMP-13, causes disruption of subchondral bone homeostasis as well as accelerates articular cartilage lesions [222]. A defect in osteocyte PLR impacts the phenotype of osteocytes and the regulation of osteoblasts and osteoclasts [221]. Fowler et al. explored the hypothesis that glucocorticoid administration inhibits enzymes of the perilacunar matrix, suppressing remodeling and causing the breakdown of the lacunar–canalicular network (LCN) early in the disease [223]. The insult of LCN manifests as decreased lacunar area, which interferes with mechanosensitive functions of osteocytes; a diminished number of osteocyte processes and impaired canalicular flow, which causes undernourishment of subchondral bone cells [97,224,225,226]. It results in osteocytes’ failure to perceive mechanical load and their proneness to secondary necrosis in a state of nutritional deprivation. The death of osteocytes causes new bone formation in both sclerostin-RANKL dependent and independent ways, with the latter being associated with the release of DAMPs during necrosis, which positively stimulates osteoblastogenesis and further bone sclerosis [227,228].

In the late stages of OA, osteocytes induce osteoblast mineralization and enhance osteoblast-mediated collagen type I synthesis [89]. According to Couchourel et al. data, during OA there is an increase in type I collagen α1 chain expression, while α2 chain expression remains stable, thus producing type I collagen, which has a lower affinity to calcium [229,230]. Moreover, the basal expression of impaired type I collagen is increased [230]. TGF-β1 is one of the key cytokines that accelerate new bone formation and angiogenesis, as well as plays an important role in mineralization, osteophyte, and fibrosis formation [214,230]. TGF-β1 in inactivated form is mobilized from the ECM during ongoing bone remodeling. While activated, it stimulates osteoblastogenesis as well as bone–cartilage interaction [214].

## 4. OA-Affected Impaired Interaction between Joint Compartments

Disturbed cellular and molecular coupling between the synovial membrane, articular cartilage, and subchondral bone promotes the progression of OA. The mutual modulation of the transcriptomes of the cells and unwanted changes in the expression of various mediators affect the stability of the whole joint.

### 4.1. Altered Interaction between the Synovium and Cartilage

Similarly, to the “osteochondal unit”, the “chondrosynovial unit” deserves just as much attention. Structures interconnected by a synovial fluid are linked molecularly. Together with the synovial membrane, synovial fluid contributes to inflammation and cartilage degradation. Housmans and his group explored the hypothesis that alterations of synovial fluid induce chondrocytes’ dedifferentiation and cartilage degeneration [231]. It is possible to detect the difference between several types of OA based on the immune cells and proteins in the synovial fluid of people with knee OA [163]. As mentioned above, synovial fluid changes its biochemical profile, accumulating various signaling factors that can serve as potential disease biomarkers. Yang and colleagues used antibody array technology to determine the downregulation of 20 proteins and the upregulation of 30 proteins in OA synovial fluid compared to healthy controls, as well as which proteins are involved in OA pathogenesis [232]. Exposure to IL-1α and TNF-α in synovial fluid, for example, modulates the collagen profile of FLSs and promotes the secretion of pro-inflammatory factors such as IL-6, IL-8, prostaglandin E2 (PGE2) and nitric oxide (NO). The expression of pro-inflammatory factors initiates the expression of MMPs and proinflammatory genes by both fibroblasts and chondrocytes, leading to cartilage degeneration [233]. Furthermore, extracellular vesicles derived from the synovial membrane activate chondrocytes through the NF-κB signaling pathway. This leads to the release of proteases degrading cartilage (MMP-9, MMP-13) and pro-inflammatory cytokines (IL-1β, IL-6, TNF-α), which are then found in synovial fluid [234].

By reciprocity, cellular and ECM particle debris in cartilage, which is induced by mechanical and biochemical stress, propagates DAMPs-associated low-grade inflammation in synovial tissue. Common DAMPs such as S100A8, S100A9, and S100A12 upregulate MMP-1, MMP-3, MMP-9, MMP-13, IL-6, ADAMTS-1, ADAMTS-4, ADAMTS-5, ADAMTS-12, VEGF gene expression perpetuating cartilage degradation [235,236,237]. Moreover, synovial cells, particularly fibroblasts, can directly alternate the articular cartilage by releasing proteases that degrade COMPs and other collagenous proteins via the Wnt/β -catenin and RUNX pathways. Furthermore, M1-polarized synovial macrophages from an OA joint negatively regulate cartilage regenerative capacity [238].

Together, altered cellular and molecular communications between the synovial membrane and articular cartilage may serve as amplifiers for the development of OA.

### 4.2. Impaired Osteochondral Communication during the Development of OA

The deterioration of subchondral bone predisposes to an abnormal distribution of mechanical forces transferred to the joint and precedes the breakdown of cartilage [89,239,240,241,242]. Prior subchondral bone damage increases the risk for cartilage loss within the same anatomical region of a knee joint by 7.5 times [243]. OA affects articular cartilage and subchondral bone as functional units [242]. Alterations in the osteochondral junction occur early in the disease course. They underline the bone–cartilage crosstalk and are pivotal in OA establishment. Pan et al. established that fluorescent dyes are actively transported from subchondral bone to calcified articular cartilage as diffusion capacity increases during the development of OA [71,86]. This is explained by intensive neoangiogenesis in subchondral bone with the later invasion of blood vessels into the osteochondral junction and cartilage [48]. In the pre- and early stages of OA, abnormal neoangiogenesis with type H vessel formation begins. It is supported by IL-6, IL-8, PGE2, TGF-β1, MMP-13, platelet-derived growth factor-BB (PDGF-BB), and vascular endothelial growth factor (VEGF) [229]. In the early stage of OA, one of the main factors for aberrant neoangiogenesis is PDGF-BB, which is secreted by mononuclear preosteoclasts in excessive amounts and acts on endothelial cells and pericytes in a paracrine fashion, as well as stimulating VEGF. VEGF mainly induces neoangiogenesis in late-stage OA [244]. The extent of type H vessels exhibits a positive association with cartilage breakdown [245]. The newly formed vasculature allows cartilage to access bone-released mediators such as TGF-β1, IGF-1, chondrolytic enzymes, bone marrow mononuclear cells that alter chondrocyte metabolism, the evolving calcification of hyaline cartilage, and osteoclast recruitment, which degrades the osteochondral junction. In turn, chondrocytes release proinflammatory cytokines, such as IL-1, which promote RANKL expression in osteoblasts and influence osteoclastogenesis. Furthermore, the expression of IL-6 and TNF-α induces osteoclastogenesis, which destabilizes the bone microenvironment [89]. New vasculature is accompanied by the novel ingrowth of nerve fibers that invade aneural cartilage tissue, causing pain [246]. This crosstalk is primarily mechanical in late-stage OA: cartilage cannot absorb excessive mechanical stress, which promotes abnormal load on the subchondral bone, aggravating remodeling [48].

### 4.3. Molecular Signaling in OA-Affected Joints

In this subsection, we have presented signaling molecules and their possible actions established between the major joint compartments—the synovial membrane, cartilage, and subchondral bone in OA-affected joints (Table 2).

## 5. Conclusions and Future Directions

Evidence from many studies on OA shows that the exact causes and effects of the disease are still not clear. Understanding the possible relationship between etiologic factors and the phenotyping of OA based on a synergy of clinical, morphological, and molecular detection methods remains one of the most challenging goals for future studies. Both the “chondrosynovial unit” and the “osteochondral unit” are essentially linked by synovial fluid; therefore, synovial fluid can be the source of important information that can be provided to assist in the creation of a personalized treatment strategy for patients. In addition, future work is needed to create a complex overview of all three joint compartments by confirming the sub-phenotypes of cells and understanding the collaboration between them at the microstructural and molecular levels. The creation of new therapeutics that alter disease-associated joint remodeling before major degeneration takes place, as well as the early identification of those who are at risk of developing OA, are two critical areas for directing future research. By achieving these two goals, we might be able to reduce the cost of OA in our society and improve the quality of life for our aging population.

## Figures and Tables

**Table 1:** The overview of signaling molecules produced by cells in the context of compartments in the healthy joint.

Chemical Messenger	Signaling Factor	Cells	Effect	References
Transcription factor	SOX-9 ^1^	Synoviocytes, chondrocytes, osteoblast lineage cells	Maintains the chondrogenic phenotype and functions of the chondrocyte, prevents hypertrophy, antagonizing with RUNX2 Inhibits IL-1β-induced inflammatory response and chondrocyte apoptosis Inhibits the production of ADAMTS in cartilage tissue	[97,109,110]
Growth factor	IGF-1 ^2^	Synoviocytes, chondrocytes, osteoblast lineage cells	Exhibits anti-inflammatory and anti-catabolic effects in chondrocytes Maintains articular cartilage anabolism, stimulating ECM production and chondrogenesis	[111,112]
Growth factor	TGF-β ^3^	Chondrocytes, synovial fibroblasts, and macrophages	Inhibits chondrocytes phenotype switch to hypertrophic chondrocytes and collagen type X production Induces proteoglycan synthesis by chondrocytes Inhibits IL-1β effects	[113]

^1^ SOX-9—SRY-box transcription factor 9; ^2^ IGF-1—insulin-like growth factor-1; ^3^ TGF-β—transforming growth factor-beta; ECM—extracellular matrix.

**Table 2:** The overview of signaling molecules produced by cells in the major joint compartments of OA-affected joint.

Chemical Messenger	Signaling Factor	Cells	Effect	References
Cytokine	IL-1β ^1^	Synovial macrophages and fibroblasts, chondrocytes, osteoblasts	Induces cartilage degradation and inhibits its repair abilities Stimulates production of MMPs (MMP-1, 3, 9, 13), ADAMTSs 4, 5 by chondrocytes Suppresses the synthesis of collagen type II and aggrecan Enhances chondrocytes’ pro-inflammatory response, hypertrophy, dedifferentiation, and apoptosis, inhibits chondrogenesis Stimulates synovial inflammation and the production of pro-inflammatory cytokines (TNF-α, IL-6, IL-8, IL-17, CCL5), mediators (NO, COX-1), prostaglandins (PGE2) Induces the formation of pannus-like tissue, fibrosis, and production of pro-fibrotic factors (PDGF, TGF-β)	[126,133,212,247,248,249]
Cytokine	IL-6	Synovial macrophages and fibroblasts, chondrocytes, osteoblasts	Promotes osteoclast formation and subchondral bone resorption Increases production of MMPs (MMP-1, 3, 13) and ADAMTS (ADAMTS-4) by chondrocytes Induces catabolic changes in chondrocytes, as well as promotes cellular senescence Shows synergic action with IL-1β and TNF-α, sustaining articular cartilage degradation and synovial inflammation	[192,250]
Cytokine	IL-17	CD4+ T cells, macrophages, NK cells, mast cells	Induces cartilage degradation by upregulating catabolic factors (MMP-1, 3, 13; ADAMTS) and downregulating anabolic factors (SOX-9, COL2A1) in chondrocytes Promotes recruitment of inflammatory cells and release of pro-inflammatory mediators, induces angiogenesis Induces RANKL expression and osteoclastogenesis	[251,252]
Cytokine	IL-18	Synovial macrophages, fibroblasts, Chondrocytes, and osteoblasts	Promotes articular cartilage degradation by upregulating MMP-1, 3, 13 and suppressing aggrecan synthesis Stimulates pro-inflammatory conditions by induction of cytokines synthesis through NF-κB and MAPK signaling pathways Stimulates both bone resorption and osteophyte formation Enhances gene expression for the synthesis of IL-6, TNF-α	[232,253,254]
Cytokine	TNF-α ^2^	Synovial macrophages and fibroblasts, chondrocytes, osteoblasts	Levels of expression are associated with radiographic OA cartilage loss Shows action synergism with IL-1β Stimulates MMP and ADAMTS production by chondrocytes; inhibits synthesis of collagen type II and aggrecan Inhibits chondrocyte differentiation by suppressing the expression of SOX-9, and induces apoptosis Promotes pro-inflammatory signaling pathways in synoviocytes and chondrocytes, stimulating the release of IL-1β, IL-6, IL-8, IL-10 Leads to neuronal sensitization, predisposing to the development of pain in OA Promotes angiogenesis and aberrant bone formation in subchondral bone by recruiting mesenchymal stem cells	[110,134,248,253,255,256,257]
Enzyme	MMPs ^3^	Chondrocytes, synovial macrophages, and fibroblasts	MMP-1 degrades collagen types I, II, III, and aggrecan of articular cartilage MMP-3 cleaves collagen types II, IV, IX, X, XI, and aggrecan; activates other MMPs (MMP-1, 7, 9) MMP-9 cleaves non-collagenous matric components MMP-13 levels correlate with hypertrophic chondrocytes in early stage OA; with OA severity and articular cartilage deterioration, as well as NF-κB expression; the enzyme exhibits higher activity for collagen type II cleavage and degrades aggrecan; it is associated with synovial membrane hyperplasia and cellular senescence	[88,133,249]
Enzyme	ADAMTSs ^4^	Chondrocytes, synovial fibroblasts, and macrophages	ADAMTS-4, 5 are induced by IL-1β, TNF-α in chondrocytes and promote cleavage of aggrecan	[206]
Transcription factor	NF-κB ^5^	All joint cells	Acts alone or in synergy with other signaling pathways Inhibits anabolic functions of chondrocytes Triggers chondrocyte hypertrophy, apoptosis, catabolic functions (MMP, ADAMTS, NO, PGE2, COX2 production), production of pro-inflammatory cytokines (IL-1β, TNF-α, IL-6, IL-8) by chondrocytes, which augment the action of NF-κB Augments activation of other transcription factors such as ELF3, and RUNX2, that stimulate MMP13 and cartilage degradation In synovial membrane promotes inflammation, angiogenesis, production of cytokines (IL-1β, TNF-α, IL-6), enzymes (MMP-1, MMP-13, ADAMTS-4, ADAMTS-5), VEGF In subchondral bone promotes resorption	[258,259,260]
Transcription factor	SOX-9 ^6^	All joint cells	Expression is downregulated in OA-affected joint Downregulation results in calcified cartilage and osteophyte formation	[110]
Growth factor	TGF-β ^7^	Chondrocytes, osteoblasts, osteoclasts, synovial fibroblasts, and macrophages	Leads to the osteoid formation and bone sclerosis Leads to cartilage damage and angiogenesis	[113]

^1^ IL—interleukin; ^2^ TNF-α—tumor necrosis factor-alpha; ^3^ MMP—matrix metalloproteinase; ^4^ ADAMTS—a disintegrin and metalloproteinase with thrombospondin motifs; ^5^ NF-κB—nuclear factor kappa B; ^6^ SOX-9—SRY-box transcription factor 9; ^7^ TGF-β—transforming growth factor-beta.

## Data Availability

A publicly available bibliographic database, PubMed.gov, was used in this study. The full bibliographic reference list is available upon request from the corresponding author.
